# Mitochondrial effectors of cellular senescence: beyond the free radical theory of aging

**DOI:** 10.1111/acel.12287

**Published:** 2014-11-14

**Authors:** Dorian V Ziegler, Christopher D Wiley, Michael C Velarde

**Affiliations:** 1 Département de Biologie, Ecole Normale Supérieure de Lyon46 allée d'Italie, Lyon, 69007, France; 2 Buck Institute for Research on Aging8001 Redwood Blvd., Novato, CA, 94945, USA

**Keywords:** aging, bioenergetics, cellular senescence, electron transport chain, metabolism, mitochondria, NAD, reactive oxygen species

## Abstract

Cellular senescence is a process that results from a variety of stresses, leading to a state of irreversible growth arrest. Senescent cells accumulate during aging and have been implicated in promoting a variety of age-related diseases. Mitochondrial stress is an effective inducer of cellular senescence, but the mechanisms by which mitochondria regulate permanent cell growth arrest are largely unexplored. Here, we review some of the mitochondrial signaling pathways that participate in establishing cellular senescence. We discuss the role of mitochondrial reactive oxygen species (ROS), mitochondrial dynamics (fission and fusion), the electron transport chain (ETC), bioenergetic balance, redox state, metabolic signature, and calcium homeostasis in controlling cellular growth arrest. We emphasize that multiple mitochondrial signaling pathways, besides mitochondrial ROS, can induce cellular senescence. Together, these pathways provide a broader perspective for studying the contribution of mitochondrial stress to aging, linking mitochondrial dysfunction and aging through the process of cellular senescence.

## Introduction

Mitochondria generate reactive oxygen species (ROS) in the form of superoxides as byproducts of the inefficient transfer of electrons across the electron transport chain (ETC) (Quinlan *et al*., 2013). Superoxide radicals can further react to form other ROS, such as hydrogen peroxides and hydroxyl radicals. These superoxides and other ROS can damage the mitochondria and further decrease the efficiency of the mitochondrial ETC, resulting in a positive feedback loop of mitochondrial ROS generation and mitochondrial oxidative damage (Balaban *et al*., 2005). For decades, this accumulation of mitochondrial oxidative damage with age has been proposed to contribute to aging and age-related phenotypes (Harman, 1956). This is the basis for the free radical theory of aging. However, while several studies support the free radical theory of aging (Melov *et al*., 2001; Kirby *et al*., 2002), other reports are now showing that increased ROS production does not always shorten lifespan (Van Raamsdonk & Hekimi, 2012), and can even promote longevity (Van Raamsdonk & Hekimi, 2009; Yee *et al*., 2014). This suggests that steady increase in ROS generation as described by the free radical theory of aging may not be sufficient to explain the phenotypes associated with aging. Hence, other factors may contribute to the aging process.

Cellular senescence, which is a biological process that causes cells to reach a state of irreversible growth arrest (Hayflick & Moorhead, 1961), may be an important factor that contributes to the aging phenotype. Indeed, senescent cells accumulate with age (Jeyapalan *et al*., 2007) and are thought to promote age-related phenotypes. Elimination of senescent cells delays age-related pathologies in a mouse model of aging (Baker *et al*., 2011). Senescent cells can contribute to aging by accelerating loss of tissue regeneration through depletion of stem cells and progenitors cells (Campisi & D'Adda di Fagagna, 2007). They also secrete several cytokines, growth factors, and proteases, collectively termed as senescence-associated secretory phenotype (SASP) (Coppé *et al*., 2008). These SASP factors have multiple autocrine and paracrine activities, which are capable of altering tissue homeostasis (Krtolica *et al*., 2001; Coppé *et al*., 2008). Hence, cellular senescence is implicated in several pathological conditions associated with aging (Campisi, 2013).

**Figure 1:**
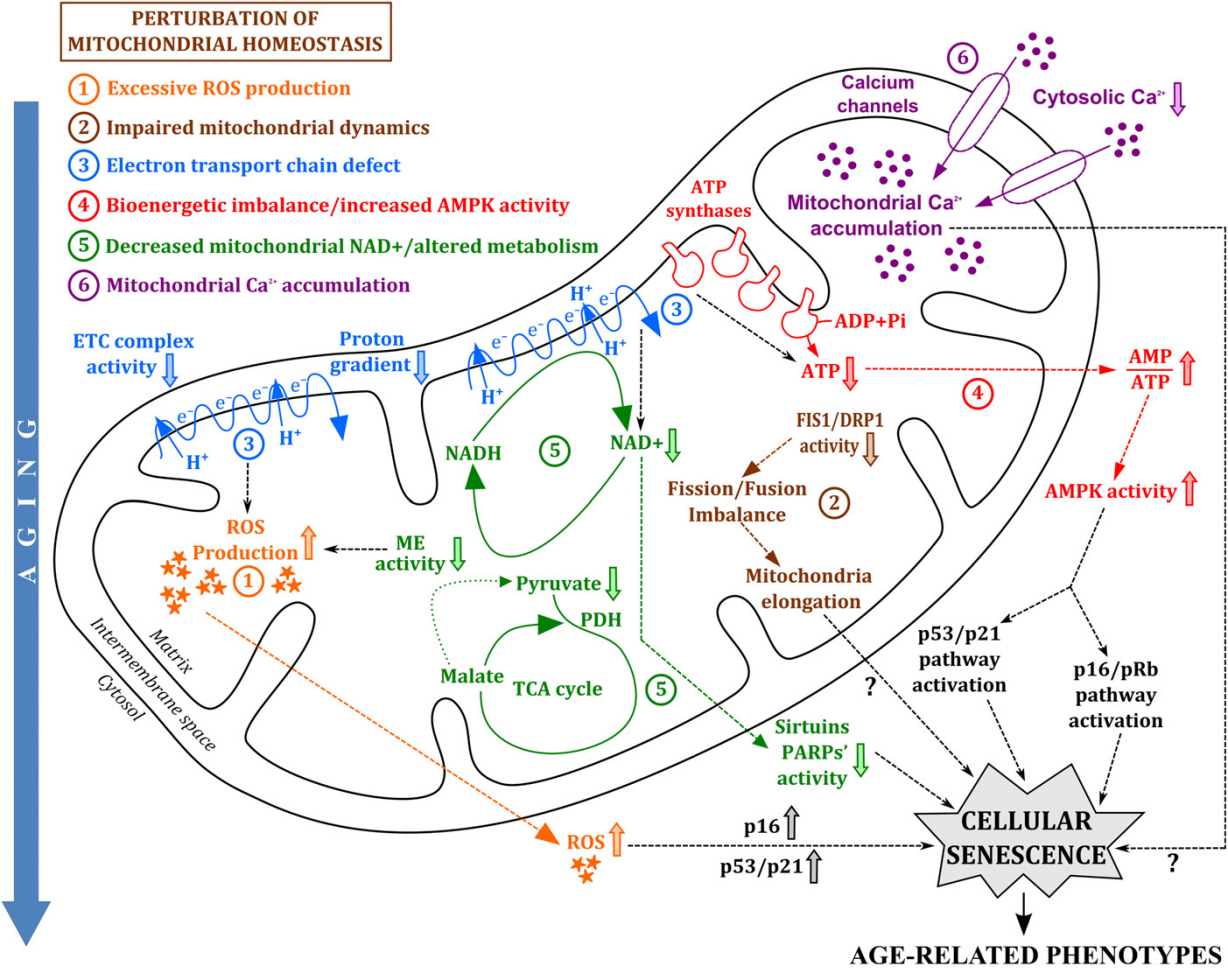
Perturbation of mitochondrial homeostasis promotes the establishment and maintenance of cellular senescence during aging. Mitochondria are damaged over time leading to perturbation of mitochondrial homeostasis. Loss of proper mitochondrial homeostasis can promote cellular senescence through (1) excessive ROS production (orange), (2) impaired mitochondrial dynamics (brown), (3) electron transport chain defect (blue), (4) bioenergetics imbalance and increased AMPK activity (red), (5) decreased mitochondrial NAD+ and altered metabolism (green), and (6) mitochondrial calcium accumulation (purple). These mitochondrial signals trigger p53/p21 and/or p16/pRb pathways and ultimately lead to cellular senescence, which subsequently promotes age-related phenotypes, such as loss of tissue regeneration and function.

Cellular senescence is accompanied by an increase in cell size (Hayflick & Moorhead, 1961), lysosomal content (Kurz *et al*., 2000), and senescence-associated β-galactosidase (SA-βgal) activity (Dimri *et al*., 1995; Kurz *et al*., 2000). It is associated with decreased nuclear expression of lamin B1 (Freund *et al*., 2012) and release of high-mobility group box 1 (HMGB1) proteins (Davalos *et al*., 2013). It is often correlated with the presence of nuclear DNA damage foci (Rodier *et al*., 2009) and chromatin alterations (Narita *et al*., 2003). It is induced by multiple factors, such as repeated cell culture, telomere attrition, irradiation, oncogene activation, and oxidative damage (Hayflick & Moorhead, 1961; Campisi & D'Adda di Fagagna, 2007). It can also be caused by the perturbation of mitochondrial homeostasis (Fig. 1), which may accelerate age-related phenotypes (Sahin & DePinho, 2010, 2012). While several studies already show that mitochondrial defects can promote cellular senescence (Passos *et al*., 2007; Moiseeva *et al*., 2009; Velarde *et al*., 2012), the mechanisms involved in this regulation are poorly understood. Because mitochondria can generate ROS (Quinlan *et al*., 2013), it is proposed that excessive mitochondrial ROS is important to establish cellular senescence. This has been an attractive model because of its consistency with the free radical theory of aging. However, other mitochondrial factors may be equally or even more important to induce cellular senescence.

In this review, we summarize the mechanisms involved in the contribution of mitochondria to senescence. In addition to mitochondrial-derived ROS production, we also discuss the role of other mitochondrial effectors, such as impaired mitochondrial dynamics, defective ETC, imbalanced bioenergetics, altered redox state, altered metabolism, and dysregulated calcium homeostasis, in establishing permanent growth arrest (Fig. 1).

## Mitochondrial free radical theory of aging and cellular senescence

The free radical theory of aging has been adapted to the study of cellular senescence. Many studies show that ROS can induce cellular senescence. Indeed, hydrogen peroxide (H_2_O_2_), which is considered as the major ROS within the cell, is a potent inducer of cellular senescence in many cell types (Ben-Porath & Weinberg, 2005). While exogenous treatment with H_2_O_2_ can promote cellular senescence, endogenous ROS (such as superoxides and hydroxyl radicals) is also implicated in the establishment and maintenance of the irreversible growth arrest. Excessive production of ROS is associated with the implementation of replicative senescence, oncogene-induced senescence, and p16^INK4A^-induced senescence (Colavitti & Finkel, 2005; Passos *et al*., 2007, 2010; Lu & Finkel, 2008; Moiseeva *et al*., 2009; Imai *et al*., 2014). A positive feedback loop of mitochondrial damage, ROS production, and DNA damage response by the activation of p53/p21^CIP1/WAF1^ pathway is required for the establishment of the growth arrest phenotype during cellular senescence (Macip *et al*., 2002; Passos *et al*., 2010; Luo *et al*., 2011). The steady increase in ROS production by this positive feedback loop is shown to replenish short-lived DNA damage foci and maintain an ongoing DNA damage response, which are thought to be both necessary and sufficient to establish and maintain cell cycle arrest during the early development of the senescence phenotype (Passos *et al*., 2010). However, this loop is no longer required to maintain the growth arrest phenotype at time points later than 9 days after initiation of senescence, suggesting that ROS production is dispensable once the senescent phenotype is fully established.

Aside from the p53/p21^CIP1/WAF1^ pathway, the p16^INK4A^/Rb pathway can also promote a ROS-dependent positive feedback loop, which reinforces the irreversible cell cycle arrest in senescent cells, partly through the downregulation of large tumor suppressor kinase 1 (LATS1), a kinase required for cytokinesis (Takahashi *et al*., 2006). Moreover, decreasing ROS levels by treatment with the mitochondria-targeted antioxidant MitoQ delays replicative senescence (Saretzki *et al*., 2003).

While several studies implicate the role of ROS during cellular senescence, others also suggest that mitochondrial ROS generation may not necessarily be the primary cause of cellular senescence. One study using an empirical mathematical model (stochastic step model of replicative senescence) suggests that increased mitochondrial ROS production in replicative senescent cells is a consequence of the senescence phenotype rather than the reverse (Lawless *et al*., 2012). Another report shows that over-expression of the mitochondrial localized antioxidant superoxide dismutase 2 (SOD2) and the mitochondrial targeted catalase are not sufficient to inhibit the senescence phenotype in hyperoxia-induced senescent cells (Klimova *et al*., 2009). Because ROS are produced by mitochondrial and nonmitochondrial enzymes during hyperoxia (70% O_2_), the inability of mitochondrial antioxidants to reverse growth arrest in hyperoxia-induced senescence suggests that cytosolic ROS may be sufficient to induce growth arrest. Hence, the mechanisms involved in linking mitochondrial ROS and cellular senescence still need to be further studied.

Because mitochondria influence many cellular processes, accumulation of mitochondrial oxidative damage, as proposed in the free radical theory of aging, may be an oversimplification of the signaling mechanisms involved in the establishment of cellular senescence. It is also possible that mitochondrial ROS can act as signaling molecules to trigger cellular senescence, independent of mitochondrial oxidative damage, although this hypothesis still needs to be proven. It is then necessary to go beyond the free radical theory and examine other mitochondrial effectors that may be involved in the irreversible cell growth arrest.

## Mitochondrial dynamics and cellular senescence

Mitochondria are known to be highly dynamic organelles. They are able to divide and combine through the process of fission and fusion, allowing them to adjust their size, shape, and organization inside the cell (Chan, 2006). Mitochondrial dynamics are regulated during cell division, apoptosis, autophagy, mitochondrial biogenesis, and mtDNA integrity maintenance (Detmer & Chan, 2007) and are implicated in aging (Seo *et al*., 2010). In mammalian cells, dynamin 1-like (DNM1L or DRP1) and fission 1 (FIS1) are involved in the fission process, while optic atrophy 1 (OPA1) and mitofusin 1 & 2 (MFN1 and MFN2) participate in the fusion process.

Altering mitochondrial dynamics can cause mitochondrial defects (Detmer & Chan, 2007; Seo *et al*., 2010), and in some cases, the implementation of cellular senescence (Jendrach *et al*., 2005; Yoon *et al*., 2006; Lee *et al*., 2007; Mai *et al*., 2010; Park *et al*., 2010; Hara *et al*., 2013). Maintenance of elongated mitochondria by blocking the fission process through FIS1 depletion leads to the establishment of senescence (Lee *et al*., 2007). Depletion of membrane-associated ring finger C3HC4 5 (MARCH5), a mitochondrial E3 ubiquitin ligase, which blocks DRP1 activity and elongates mitochondria, also induces senescence (Park *et al*., 2010). These data suggest that senescent cells are typically associated with an overall shift toward more fusion events, resulting in the presence of abnormally enlarged mitochondria.

While studies correlate elongated mitochondria with the establishment of cellular senescence, it is still unclear how mitochondrial fusion contributes to the permanent cell growth arrest phenotype or whether mitochondrial fusion is merely a response to cellular stress. Some studies show that prolonged elongated mitochondria result in higher production of intracellular ROS and lower activity of mitochondrial respiration, which then ultimately leads to cellular senescence (Yoon *et al*., 2006). However, others suggest that mitochondrial fusion may protect a cell from excessive mitochondrial stress by maintaining a functional population of mitochondria within a cell (Chen *et al*., 2005). Mitochondrial fusion, in response to cellular stress, allows mitochondria to possess more cristae, stimulate more ATP synthase activity, maintain ATP production, and escape autophagic degradation (Gomes *et al*., 2011). Moreover, increasing mitochondrial fusion also prevents mitochondrial membrane depolarization, inhibits cytochrome c release, and promotes resistance to apoptosis (Frank *et al*., 2001; Karbowski *et al*., 2002; Beckenridge *et al*., 2003; Brooks *et al*., 2009). Hence, mitochondrial fusion can also provide a way for defective mitochondria to restore their essential components and regain their cellular function. Whether this pro-survival response after mitochondrial stress predisposes cells to senesce instead of apoptose remains to be determined.

There are still many remaining questions regarding the role of mitochondrial dynamics and cellular senescence. It is still unknown whether changes in mitochondrial morphology play a significant role in establishing senescence or whether these changes are merely a consequence of the process. Nonetheless, current data do suggest that changes in mitochondrial dynamics can promote cellular senescence (Fig. 1). Elucidating the consequences of prolonged elongated mitochondria on cell signaling and cellular function may help determine the mechanisms involved in cell growth arrest following mitochondrial stress.

## Mitochondrial electron transport chain and cellular senescence

In addition to altered mitochondrial dynamics, damage to the mitochondrial ETC is also a form of mitochondrial stress, shown to cause cellular senescence (Fig. 1). Indeed, pharmacological inhibition and genetic loss of function of the ETC can lead to premature senescence. Inhibition of complex I by rotenone or of complex II by 2-thenoyltrifluoroacetone (TFFA) induces cellular senescence (Yoon *et al*., 2003; Moiseeva *et al*., 2009). Similarly, knockdown of the mitochondrial Rieske iron-sulfur polypeptide (RISP), which is involved in the transport of electrons to complex III, also drives senescence in human fibroblasts (Moiseeva *et al*., 2009). Inhibition of mitochondrial complex III by antimycin A also promotes a cell proliferation arrest and premature senescence, as evident by upregulation of the cyclin-dependent kinase inhibitors p16^INK4A^ and p21^CIP1/WAF1^ (Stöckl *et al*., 2006).

The Mitochondrial ETC requires a proton gradient across the mitochondrial membrane to function (Saraste, 1999). Hence, mitochondrial depolarization stalls the mitochondrial ETC and promotes mitochondrial defect. Interestingly, loss of this proton gradient by uncouplers such as carbonylcyanide-p-trifluoromethoxyphenylhydrazone (FCCP) also induces cellular senescence in human fibroblasts (Stöckl *et al*., 2007), further supporting the consequence of defective mitochondrial ETC to establish cellular senescence.

While several evidences show that inhibition of the ETC leads to cellular senescence, the specific signaling pathway linking the ETC defect and growth arrest is still unclear. One speculation is that impaired ETC can increase ROS production and promote mitochondrial damage, which then results in cellular senescence. However, this hypothesis still needs to be critically tested.

Studies suggest that there is an age-dependent decrease in the ETC. *In vivo*, such as those in flies (McCarroll *et al*., 2004; Ferguson *et al*., 2005), worms (McCarroll *et al*., 2004), and monkeys (Kayo *et al*., 2001), commonly through a downregulation of genes involved in the ETC function. Agents such as mitochondrial oxidative damage, mitochondrial DNA mutation, and environmental factors can all damage the ETC (Li *et al*., 1995; Vermulst *et al*., 2008; Krutmann & Schroeder, 2009), but which of these factors is the primary driver of ETC defects *in vivo* remains debatable. Further research needs to be performed to identify the specific pathway most relevant during the aging process.

## Mitochondrial bioenergetic balance and cellular senescence

The mitochondrial ETC produces ATP as an important source of cellular energy during aerobic respiration. Defects in the ETC lead to a drop in ATP production and can result in the induction of cellular senescence (Fig. 1). Indeed, inhibition of ATP synthesis triggers senescence, as observed by upregulation of p16^INK4A^ and p21^CIP1/WAF1^ expression (Stöckl *et al*., 2006). Decrease in ATP production can also increase AMP (or ADP) to ATP ratio, creating a bioenergetic imbalance within the cell. Interestingly, some reports do show an increased AMP to ATP ratio during cellular senescence (Wang *et al*., 2003; Zwerschke *et al*., 2003). Elevation of AMP to ATP ratios by depleting ATP levels or by addition of exogenous AMP promotes cellular growth arrest and senescence features (Zwerschke *et al*., 2003).

Increased AMP (or ADP) to ATP ratios stimulate AMP-activated protein kinase (AMPK), which is known to be a central mediator of cellular metabolism in eukaryotes (Mihaylova & Shaw, 2011). AMPK activation induces cell cycle arrest in many cells, including mouse embryonic cells (MEFs), human fibroblasts, human cancer cells, and fly eye cells (Jones *et al*., 2005; Rattan *et al*., 2005; Owusu-Ansah *et al*., 2008; Humbert *et al*., 2010; Mandal *et al*., 2010; Hou *et al*., 2011; Peyton *et al*., 2012). Multiple distinct AMPK-related mechanisms have been described in establishing and maintaining cellular senescence (Fig. 1). One mechanism involves an AMPK-dependent pathway and the other an AMPK-related protein kinase 5 (ARK5 or NUAK1)-dependent pathway. Persistent activation of AMPK increases p53 expression and phosphorylation, upregulates p21^CIP1/WAF1^ and p27 expression (Peyton *et al*., 2012), and promotes a p53-dependent senescence (Jones *et al*., 2005; Jiang *et al*., 2013). Activated AMPK also induces cell cycle arrest by downregulating pro-proliferation genes, such as cyclin A, cyclin B1, and cyclin E (Wang *et al*., 2002, 2003; Mandal *et al*., 2010; Peyton *et al*., 2012). AMPK also inhibits the RNA-stabilizing factor human antigen R (HuR), which destabilizes p16^INK4A^, leading to increased p16^INK4A^ expression and ultimately to senescence (Wang *et al*., 2002, 2003). AMPK activation reduces retinoblastoma protein phosphorylation (Peyton *et al*., 2012), leading to the inhibition of cell proliferation. Furthermore, activation of the AMPK-related protein ARK5 promotes senescence either through a p53/p21^CIP1/WAF1^-dependent pathway (Hou *et al*., 2011) or through a p53-independent LATS1-dependent pathway (Humbert *et al*., 2010).

AMPK activity is highly increased in oncogene-induced senescent cells (Moiseeva *et al*., 2009). In contrast, inactivation of the AMPK pathway is known to promote cancer (Bardeesy *et al*., 2002; Huang *et al*., 2008b; Shackelford & Shaw, 2009; Zhou *et al*., 2009), further supporting the role of AMPK in establishing growth arrest and tumor suppression. Hence, studies emphasizing the impact of mitochondrial bioenergetic balance and subsequent AMPK activation may provide insights into the mechanisms involved in establishing cellular senescence and their contribution to aging and age-related phenotypes.

## Mitochondrial metabolites and cellular senescence

Protein complexes in the mitochondrial ETC produce important cofactors and metabolites necessary for cellular function. Complex I of the ETC oxidizes the reduced form of nicotinamide adenine dinucleotide (NADH) into NAD+, which is a cofactor of many intracellular enzymes. Interestingly, depletion of NAD+ is implicated in cellular senescence (Fig. 1). Indeed, impaired NAD+ salvage pathway induces premature senescence, while activation of the NAD+ salvage pathway extends cellular replicative lifespan (van der Veer *et al*., 2007; Borradaile & Pickering, 2009; Ho *et al*., 2009). Furthermore, depletion of cytosolic malate dehydrogenase (MDH1), a key component in the malate–aspartate shuttle, which transfers reducing equivalents of NADH across the inner mitochondrial membrane, results in a decrease in NAD+/NADH ratio, AMPK activation, and cellular senescence (Lee *et al*., 2012). Increased NAD+/NADH ratios seem to limit oxidative stress by enhancing aerobic glycolysis, which supports proliferation while limiting ROS production (Borradaile & Pickering, 2009).

NAD+ is required for many enzymatic reactions, such as those involved in glycolysis, the tricarboxylic acid (TCA) cycle, DNA repair, and protein acetylation. For example, NAD+ is essential for the activities of poly-ADP ribose polymerases (PARPs), which are important for DNA repair and for the activities of sirtuins, which constitute a class of protein deacetylases implicated in aging and longevity (Longo & Kennedy, 2006; Haigis & Sinclair, 2010). PARPs and sirtuins are known to play roles in cellular senescence. PARPs prevent cellular senescence by promoting repair of DNA strand breaks in response to genotoxic stress (Efimova *et al*., 2010). Sirtuin 1 antagonizes senescence by deacetylating p53 in MEFs (Langley *et al*., 2002), activating ERK/S6K1 signaling pathway in human fibroblasts (Huang *et al*., 2008a), and preventing LKB1 dependent-AMPK activation in porcine endothelial cells (Zu *et al*., 2010). Another NAD+-dependent protein implicated in senescence is malic enzyme, which converts malate into pyruvate. Depletion of the mitochondrial NAD(P)+-dependent malic enzyme (ME2) triggers p53-dependent senescence by increasing ROS level and activating AMPK (Jiang *et al*., 2013). Taken together, these data indicate that mitochondrial depletion of NAD+ levels and subsequent decreased activity of NAD+-dependent enzymes can ultimately lead to cellular senescence.

Besides NAD+, other metabolites are also produced in the mitochondria. Several metabolic intermediates of the TCA cycle can influence cellular function and potentially contribute to aging and age-related phenotypes (Salminen *et al*., 2014). Senescent cells are associated with altered metabolism, such as decreased aerobic glycolysis, increased alanine production, decreased overall ribonucleotide triphosphate content (Wang *et al*., 2003; Zwerschke *et al*., 2003), reduced lipid synthesis, and enhanced fatty acid oxidation (Quijano *et al*., 2012), but whether these changes are causes or consequences of cellular senescence remains unclear. One study suggests that increasing pyruvate consumption and cellular respiration by overexpression of the mitochondrial enzyme pyruvate dehydrogenase (PDH) enhances BRAF^V600E^ oncogene-induced senescence (Kaplon *et al*., 2013). Another study reveals that senescence-associated telomere dysfunction is sufficient to perturb mitochondrial function, through a p53-dependent downregulation of the mitochondrial master regulators such as peroxisome proliferator-activated receptor gamma, coactivator one alpha and beta (Sahin *et al*., 2011).

While several reports implicate the role of mitochondrial metabolites in establishing senescence, the particular mechanism on how these cofactors can promote cell cycle arrest remains elusive. Because studies regarding the role of the different metabolites on cellular senescence are limited, understanding the metabolic signature of senescent cells and whether this altered metabolic profile contributes to permanent cell cycle arrest are important avenues to explore in future.

## Mitochondrial calcium homeostasis and cellular senescence

As mentioned above, mitochondria require a proton gradient across their membrane for the ETC to function. Disrupting this mitochondrial membrane potential can result in decreased ATP production and cellular senescence. Mitochondria import calcium to maintain ETC function and intracellular calcium homeostasis (Gunter *et al*., 2004), but as a consequence of increased uptake, this also depolarizes the mitochondria, decreases overall ATP production (Nguyen & Jafri, 2005), elevates cytosolic NADH, and reduces sirtuin activity (Marcu *et al*., 2014), which can all lead to cellular senescence (Fig. 1). Indeed, accumulation of mitochondrial calcium is implicated in oncogene-induced senescence and replicative senescence (Bolinches-Amorós *et al*., 2014; Wiel *et al*., 2014, 2014). Consistently, loss of functional calcium channels, such as mitochondrial calcium uniporter (MCU), prevents calcium uptake by the mitochondria, resulting in the escape from oncogene-induced senescence (Wiel *et al*., 2014).

While the accumulation of calcium in the mitochondria disrupts mitochondrial membrane potential, mitochondria do maintain low levels of calcium and act as intracellular calcium reservoirs (Butow & Avadhani, 2004). In response to stress, mitochondria can release these calcium ions and trigger a retrograde response, which signals to the nucleus and activates specific nuclear transcription factors (Butow & Avadhani, 2004). One of these transcription factors, cAMP-responsive element binding protein 1 (CREB), upregulates p21^CIP1/WAF1^ expression and acts as a potent inhibitor of cell proliferation (Arnould *et al*., 2002). While the retrograde response is an attractive mechanism for mitochondrial stress-induced senescence, this hypothesis needs to be further confirmed. Understanding the contribution of calcium homeostasis to permanent cell cycle arrest may potentially offer new perspectives in linking mitochondrial stress and cellular senescence.

## Conclusion

In this review, we report the different mechanisms by which mitochondria contribute to the implementation of cellular senescence. We propose a model where the mitochondrion acts as a key player in promoting and establishing permanent growth arrest. We suggest that perturbation of mitochondrial homeostasis triggers cellular senescence, which can ultimately lead to age-associated pathologies (Fig. 1). Multiple mitochondrial factors, such as excessive mitochondrial ROS production, aberrant mitochondrial dynamics, defective electron transport chain, imbalanced bioenergetics, activated AMPK, decreased NAD+ levels, altered metabolism, and dysregulated mitochondrial calcium homeostasis, contribute to the establishment of irreversible growth arrest (Fig. 1). All of these different mitochondrial signaling pathways can regulate each other, but how these factors cooperate to promote cellular senescence, and whether these pathways are conserved in all senescent cells still remains unclear. Nonetheless, studying these different factors can provide new insights into the mechanisms involved in mitochondrial dysfunction-associated senescence. Because both mitochondrial defects and cellular senescence accumulate with age, linking the pathways involved in these two phenomena may help us understand the biology of aging, providing new potential targets to treat age-related diseases.
